# Supplementary material for: Modeling glioblastoma heterogeneity as a dynamic network of cell states
Source: Mol Syst Biol. 2021 Sep 16;17(9):e10105. doi: 10.15252/msb.202010105 (PMC8444284; doi:10.15252/msb.202010105)
Supplement: Supplementary file 5 — Source Data for Figure 3 [file MSB-17-e10105-s001.zip › Figure3A_sourcedata/GSEA_3065/nosigdb_state5.GseaPreranked.1622718435914/FELDMAN_2020_NEURAL G0.html]

Details for gene set FELDMAN\_2020\_NEURAL G0[GSEA]

|  || Dataset | state5 |
| Phenotype | NoPhenotypeAvailable |
| Upregulated in class | na\_neg |
| GeneSet | FELDMAN\_2020\_NEURAL G0 |
| Enrichment Score (ES) | -0.47929382 |
| Normalized Enrichment Score (NES) | -1.8597008 |
| Nominal p-value | 0.0 |
| FDR q-value | 8.982762E-4 |
| FWER p-Value | 0.021 |
Table: GSEA Results Summary

  

Fig 1: Enrichment plot: FELDMAN\_2020\_NEURAL G0      
 Profile of the Running ES Score & Positions of GeneSet Members on the Rank Ordered List

  

| PROBE | GENE SYMBOL | GENE\_TITLE | RANK IN GENE LIST | RANK METRIC SCORE | RUNNING ES | CORE ENRICHMENT || 1 | MALAT1 |  |  | 0 | 1.196 | 0.0102 | No |
| 2 | NOVA1 |  |  | 10 | 0.870 | 0.0166 | No |
| 3 | PTPRZ1 |  |  | 12 | 0.862 | 0.0238 | No |
| 4 | WSB1 |  |  | 13 | 0.861 | 0.0312 | No |
| 5 | PTN |  |  | 19 | 0.824 | 0.0376 | No |
| 6 | SOX6 |  |  | 28 | 0.754 | 0.0431 | No |
| 7 | HSP90B1 |  |  | 33 | 0.729 | 0.0489 | No |
| 8 | SCRG1 |  |  | 36 | 0.715 | 0.0548 | No |
| 9 | PLAT |  |  | 37 | 0.714 | 0.0609 | No |
| 10 | SQLE |  |  | 39 | 0.705 | 0.0668 | No |
| 11 | GPM6B |  |  | 41 | 0.702 | 0.0726 | No |
| 12 | LAPTM4A |  |  | 51 | 0.641 | 0.0771 | No |
| 13 | GPM6A |  |  | 60 | 0.609 | 0.0813 | No |
| 14 | LAMP2 |  |  | 69 | 0.584 | 0.0854 | No |
| 15 | PON2 |  |  | 84 | 0.546 | 0.0884 | No |
| 16 | FGFBP3 |  |  | 85 | 0.545 | 0.0931 | No |
| 17 | LRRC4C |  |  | 101 | 0.525 | 0.0958 | No |
| 18 | CADM4 |  |  | 102 | 0.525 | 0.1003 | No |
| 19 | PRCP |  |  | 103 | 0.523 | 0.1048 | No |
| 20 | ITM2B |  |  | 117 | 0.509 | 0.1076 | No |
| 21 | SCARB2 |  |  | 134 | 0.494 | 0.1099 | No |
| 22 | TTYH1 |  |  | 141 | 0.489 | 0.1134 | No |
| 23 | COL9A3 |  |  | 144 | 0.489 | 0.1174 | No |
| 24 | EFHC1 |  |  | 148 | 0.486 | 0.1212 | No |
| 25 | TMEM59 |  |  | 151 | 0.481 | 0.1250 | No |
| 26 | CLU |  |  | 162 | 0.474 | 0.1279 | No |
| 27 | SCG2 |  |  | 166 | 0.470 | 0.1316 | No |
| 28 | ASAH1 |  |  | 172 | 0.462 | 0.1349 | No |
| 29 | SCD5 |  |  | 173 | 0.460 | 0.1389 | No |
| 30 | GGH |  |  | 184 | 0.453 | 0.1416 | No |
| 31 | CTSV |  |  | 193 | 0.443 | 0.1444 | No |
| 32 | SLC44A1 |  |  | 216 | 0.428 | 0.1455 | No |
| 33 | PSAP |  |  | 227 | 0.420 | 0.1480 | No |
| 34 | MFGE8 |  |  | 233 | 0.418 | 0.1509 | No |
| 35 | SERPINE2 |  |  | 238 | 0.414 | 0.1540 | No |
| 36 | ARL6IP5 |  |  | 256 | 0.406 | 0.1555 | No |
| 37 | CDH2 |  |  | 261 | 0.403 | 0.1585 | No |
| 38 | SYT11 |  |  | 265 | 0.400 | 0.1615 | No |
| 39 | SPARC |  |  | 327 | 0.370 | 0.1576 | No |
| 40 | SGCB |  |  | 328 | 0.370 | 0.1608 | No |
| 41 | CNPY2 |  |  | 336 | 0.365 | 0.1631 | No |
| 42 | NDFIP1 |  |  | 386 | 0.348 | 0.1604 | No |
| 43 | LINC00461 |  |  | 417 | 0.335 | 0.1597 | No |
| 44 | IGFBP7 |  |  | 424 | 0.333 | 0.1619 | No |
| 45 | BCHE |  |  | 425 | 0.333 | 0.1647 | No |
| 46 | DNAJC1 |  |  | 517 | 0.306 | 0.1568 | No |
| 47 | EDNRB |  |  | 519 | 0.306 | 0.1593 | No |
| 48 | MAP2 |  |  | 549 | 0.298 | 0.1584 | No |
| 49 | SSR3 |  |  | 575 | 0.291 | 0.1580 | No |
| 50 | PLOD2 |  |  | 613 | 0.282 | 0.1561 | No |
| 51 | ZKSCAN1 |  |  | 678 | 0.269 | 0.1510 | No |
| 52 | SPP1 |  |  | 724 | 0.256 | 0.1480 | No |
| 53 | RNF13 |  |  | 745 | 0.251 | 0.1478 | No |
| 54 | DHCR24 |  |  | 795 | 0.242 | 0.1442 | No |
| 55 | ORC6 |  |  | 806 | 0.240 | 0.1451 | No |
| 56 | PRSS23 |  |  | 831 | 0.235 | 0.1443 | No |
| 57 | NT5E |  |  | 834 | 0.235 | 0.1460 | No |
| 58 | DCBLD2 |  |  | 863 | 0.230 | 0.1448 | No |
| 59 | DLG1 |  |  | 924 | 0.221 | 0.1397 | No |
| 60 | LAMA4 |  |  | 931 | 0.220 | 0.1409 | No |
| 61 | IFI6 |  |  | 961 | 0.216 | 0.1393 | No |
| 62 | FCGRT |  |  | 971 | 0.214 | 0.1401 | No |
| 63 | ASPM |  |  | 980 | 0.213 | 0.1410 | No |
| 64 | PLP1 |  |  | 1022 | 0.208 | 0.1380 | No |
| 65 | EBP |  |  | 1041 | 0.206 | 0.1377 | No |
| 66 | PRKDC |  |  | 1051 | 0.204 | 0.1384 | No |
| 67 | BAMBI |  |  | 1144 | 0.192 | 0.1294 | No |
| 68 | EIF4A2 |  |  | 1153 | 0.191 | 0.1301 | No |
| 69 | PCSK1N |  |  | 1162 | 0.190 | 0.1307 | No |
| 70 | DHRS7 |  |  | 1239 | 0.181 | 0.1235 | No |
| 71 | IFITM3 |  |  | 1332 | 0.168 | 0.1142 | No |
| 72 | DKK1 |  |  | 1388 | 0.163 | 0.1092 | No |
| 73 | SRPX |  |  | 1409 | 0.161 | 0.1083 | No |
| 74 | CLDND1 |  |  | 1501 | 0.152 | 0.0990 | No |
| 75 | CENPF |  |  | 1531 | 0.149 | 0.0969 | No |
| 76 | TMEFF2 |  |  | 1546 | 0.147 | 0.0965 | No |
| 77 | PLD3 |  |  | 1562 | 0.145 | 0.0960 | No |
| 78 | CST3 |  |  | 1602 | 0.141 | 0.0927 | No |
| 79 | MOXD1 |  |  | 1639 | 0.139 | 0.0897 | No |
| 80 | CYR61 |  |  | 1850 | 0.123 | 0.0664 | No |
| 81 | HNRNPA2B1 |  |  | 1852 | 0.122 | 0.0673 | No |
| 82 | KIF20B |  |  | 1962 | 0.114 | 0.0556 | No |
| 83 | ECE1 |  |  | 2044 | 0.109 | 0.0472 | No |
| 84 | TSPAN6 |  |  | 2087 | 0.106 | 0.0432 | No |
| 85 | FRMD4A |  |  | 2196 | 0.098 | 0.0315 | No |
| 86 | SDF2L1 |  |  | 2275 | 0.093 | 0.0232 | No |
| 87 | MDK |  |  | 2458 | 0.082 | 0.0028 | No |
| 88 | AKAP12 |  |  | 2478 | 0.081 | 0.0013 | No |
| 89 | RNASEH2A |  |  | 2560 | 0.076 | -0.0075 | No |
| 90 | TMEM237 |  |  | 2680 | 0.069 | -0.0207 | No |
| 91 | PLAUR |  |  | 2826 | 0.061 | -0.0370 | No |
| 92 | FDPS |  |  | 2849 | 0.060 | -0.0390 | No |
| 93 | AURKB |  |  | 2853 | 0.060 | -0.0389 | No |
| 94 | SRSF2 |  |  | 2908 | 0.057 | -0.0446 | No |
| 95 | COL11A1 |  |  | 2950 | 0.055 | -0.0489 | No |
| 96 | INSIG1 |  |  | 3055 | 0.049 | -0.0606 | No |
| 97 | SRSF7 |  |  | 3126 | 0.046 | -0.0683 | No |
| 98 | IGFBP5 |  |  | 3230 | 0.040 | -0.0799 | No |
| 99 | TIMP4 |  |  | 3284 | 0.037 | -0.0858 | No |
| 100 | TPST2 |  |  | 3303 | 0.036 | -0.0875 | No |
| 101 | SMC2 |  |  | 3374 | 0.032 | -0.0954 | No |
| 102 | PLAU |  |  | 3407 | 0.031 | -0.0988 | No |
| 103 | ESCO2 |  |  | 3672 | 0.021 | -0.1293 | No |
| 104 | LRRN3 |  |  | 3720 | 0.019 | -0.1346 | No |
| 105 | GTSE1 |  |  | 3738 | 0.018 | -0.1364 | No |
| 106 | DLGAP5 |  |  | 3907 | 0.011 | -0.1558 | No |
| 107 | ARMCX3 |  |  | 3970 | 0.008 | -0.1629 | No |
| 108 | TSPAN9 |  |  | 3977 | 0.008 | -0.1636 | No |
| 109 | LOXL1 |  |  | 3988 | 0.007 | -0.1647 | No |
| 110 | SAT2 |  |  | 4006 | 0.007 | -0.1666 | No |
| 111 | ARL5A |  |  | 4160 | 0.000 | -0.1843 | No |
| 112 | CNTN1 |  |  | 4200 | -0.001 | -0.1889 | No |
| 113 | MAGED2 |  |  | 4228 | -0.002 | -0.1920 | No |
| 114 | PRC1 |  |  | 4241 | -0.003 | -0.1933 | No |
| 115 | SOX2 |  |  | 4271 | -0.005 | -0.1967 | No |
| 116 | NUDT4 |  |  | 4430 | -0.011 | -0.2149 | No |
| 117 | CIRBP |  |  | 4617 | -0.019 | -0.2363 | No |
| 118 | NUF2 |  |  | 4628 | -0.019 | -0.2373 | No |
| 119 | CDK1 |  |  | 4766 | -0.025 | -0.2530 | No |
| 120 | CENPE |  |  | 4787 | -0.025 | -0.2551 | No |
| 121 | FAM181B |  |  | 4789 | -0.025 | -0.2550 | No |
| 122 | SPRY1 |  |  | 4844 | -0.027 | -0.2611 | No |
| 123 | TROAP |  |  | 4869 | -0.028 | -0.2636 | No |
| 124 | MCM7 |  |  | 4986 | -0.032 | -0.2768 | No |
| 125 | GINS2 |  |  | 5024 | -0.034 | -0.2808 | No |
| 126 | BTG1 |  |  | 5087 | -0.037 | -0.2877 | No |
| 127 | CCNB1 |  |  | 5111 | -0.037 | -0.2900 | No |
| 128 | CA2 |  |  | 5139 | -0.038 | -0.2929 | No |
| 129 | PDHX |  |  | 5153 | -0.038 | -0.2940 | No |
| 130 | SPC25 |  |  | 5312 | -0.045 | -0.3120 | No |
| 131 | CCNB2 |  |  | 5382 | -0.048 | -0.3196 | No |
| 132 | NUSAP1 |  |  | 5389 | -0.048 | -0.3199 | No |
| 133 | CLSPN |  |  | 5614 | -0.058 | -0.3454 | No |
| 134 | CYB5B |  |  | 5644 | -0.060 | -0.3482 | No |
| 135 | CCNA2 |  |  | 5671 | -0.061 | -0.3507 | No |
| 136 | QKI |  |  | 5715 | -0.062 | -0.3552 | No |
| 137 | TOP2A |  |  | 5722 | -0.063 | -0.3554 | No |
| 138 | CDCA3 |  |  | 5726 | -0.063 | -0.3552 | No |
| 139 | RMI2 |  |  | 5797 | -0.065 | -0.3627 | No |
| 140 | KPNA2 |  |  | 5811 | -0.066 | -0.3637 | No |
| 141 | ALDOA |  |  | 5909 | -0.070 | -0.3743 | No |
| 142 | KNSTRN |  |  | 5923 | -0.070 | -0.3753 | No |
| 143 | YPEL5 |  |  | 5945 | -0.072 | -0.3771 | No |
| 144 | SHISA4 |  |  | 5957 | -0.072 | -0.3777 | No |
| 145 | EXOSC8 |  |  | 6020 | -0.075 | -0.3843 | No |
| 146 | ATP6V0E1 |  |  | 6138 | -0.082 | -0.3972 | No |
| 147 | FHL2 |  |  | 6169 | -0.083 | -0.4000 | No |
| 148 | C1orf21 |  |  | 6205 | -0.085 | -0.4033 | No |
| 149 | MKI67 |  |  | 6211 | -0.086 | -0.4031 | No |
| 150 | RRM1 |  |  | 6298 | -0.090 | -0.4123 | No |
| 151 | RRM2 |  |  | 6308 | -0.091 | -0.4126 | No |
| 152 | CENPM |  |  | 6368 | -0.093 | -0.4187 | No |
| 153 | CKAP2 |  |  | 6372 | -0.094 | -0.4182 | No |
| 154 | CENPA |  |  | 6374 | -0.094 | -0.4175 | No |
| 155 | ZWINT |  |  | 6453 | -0.099 | -0.4257 | No |
| 156 | DNMT1 |  |  | 6455 | -0.099 | -0.4250 | No |
| 157 | SMC4 |  |  | 6517 | -0.102 | -0.4312 | No |
| 158 | KIF4A |  |  | 6529 | -0.103 | -0.4316 | No |
| 159 | VOPP1 |  |  | 6570 | -0.106 | -0.4354 | No |
| 160 | OLIG2 |  |  | 6595 | -0.108 | -0.4372 | No |
| 161 | MAD2L1 |  |  | 6600 | -0.108 | -0.4368 | No |
| 162 | HSP90AB1 |  |  | 6611 | -0.109 | -0.4370 | No |
| 163 | LMNB1 |  |  | 6638 | -0.110 | -0.4391 | No |
| 164 | MORC4 |  |  | 6639 | -0.110 | -0.4381 | No |
| 165 | PAPSS1 |  |  | 6640 | -0.110 | -0.4372 | No |
| 166 | VRK1 |  |  | 6651 | -0.111 | -0.4374 | No |
| 167 | RPAIN |  |  | 6664 | -0.112 | -0.4378 | No |
| 168 | CEP55 |  |  | 6700 | -0.114 | -0.4409 | No |
| 169 | DEPDC1 |  |  | 6723 | -0.115 | -0.4425 | No |
| 170 | TAGLN |  |  | 6733 | -0.116 | -0.4426 | No |
| 171 | LIMCH1 |  |  | 6767 | -0.119 | -0.4454 | No |
| 172 | CCDC34 |  |  | 6826 | -0.123 | -0.4511 | No |
| 173 | ANLN |  |  | 6829 | -0.123 | -0.4502 | No |
| 174 | SLC9A3R2 |  |  | 6834 | -0.124 | -0.4497 | No |
| 175 | PFKP |  |  | 6845 | -0.124 | -0.4498 | No |
| 176 | FLNA |  |  | 6856 | -0.126 | -0.4498 | No |
| 177 | PMAIP1 |  |  | 6872 | -0.128 | -0.4505 | No |
| 178 | TRAF4 |  |  | 6895 | -0.129 | -0.4520 | No |
| 179 | SESN3 |  |  | 6935 | -0.132 | -0.4554 | No |
| 180 | HSP90AA1 |  |  | 6962 | -0.134 | -0.4572 | No |
| 181 | MMAB |  |  | 6963 | -0.134 | -0.4561 | No |
| 182 | PLP2 |  |  | 6975 | -0.135 | -0.4562 | No |
| 183 | CDC20 |  |  | 6985 | -0.136 | -0.4561 | No |
| 184 | AURKA |  |  | 6990 | -0.136 | -0.4554 | No |
| 185 | TLE1 |  |  | 7005 | -0.137 | -0.4559 | No |
| 186 | ARHGAP29 |  |  | 7007 | -0.138 | -0.4548 | No |
| 187 | PBK |  |  | 7010 | -0.138 | -0.4539 | No |
| 188 | TXNIP |  |  | 7037 | -0.140 | -0.4557 | No |
| 189 | DNAJC9 |  |  | 7043 | -0.141 | -0.4551 | No |
| 190 | SH3BP5 |  |  | 7076 | -0.143 | -0.4575 | No |
| 191 | MGLL |  |  | 7100 | -0.145 | -0.4590 | No |
| 192 | C4orf48 |  |  | 7110 | -0.146 | -0.4588 | No |
| 193 | MIS18A |  |  | 7115 | -0.147 | -0.4580 | No |
| 194 | CENPW |  |  | 7116 | -0.147 | -0.4567 | No |
| 195 | RPL22L1 |  |  | 7150 | -0.150 | -0.4593 | No |
| 196 | GMNN |  |  | 7224 | -0.158 | -0.4664 | No |
| 197 | MYH9 |  |  | 7248 | -0.161 | -0.4677 | No |
| 198 | LINC00511 |  |  | 7249 | -0.162 | -0.4663 | No |
| 199 | EIF3E |  |  | 7253 | -0.162 | -0.4653 | No |
| 200 | IGBP1 |  |  | 7273 | -0.164 | -0.4661 | No |
| 201 | SMIM14 |  |  | 7281 | -0.165 | -0.4655 | No |
| 202 | TMPO |  |  | 7296 | -0.167 | -0.4657 | No |
| 203 | IDI1 |  |  | 7337 | -0.172 | -0.4689 | No |
| 204 | CRIPT |  |  | 7341 | -0.173 | -0.4677 | No |
| 205 | HEY1 |  |  | 7370 | -0.177 | -0.4695 | No |
| 206 | SNRPA1 |  |  | 7376 | -0.178 | -0.4685 | No |
| 207 | TK1 |  |  | 7377 | -0.178 | -0.4670 | No |
| 208 | RGS20 |  |  | 7387 | -0.180 | -0.4665 | No |
| 209 | TUBB6 |  |  | 7396 | -0.180 | -0.4659 | No |
| 210 | USP1 |  |  | 7398 | -0.181 | -0.4645 | No |
| 211 | APIP |  |  | 7452 | -0.187 | -0.4691 | No |
| 212 | TCEAL8 |  |  | 7456 | -0.187 | -0.4678 | No |
| 213 | NMB |  |  | 7463 | -0.187 | -0.4669 | No |
| 214 | POPDC3 |  |  | 7479 | -0.189 | -0.4670 | No |
| 215 | PLEKHB1 |  |  | 7506 | -0.191 | -0.4684 | No |
| 216 | VGLL4 |  |  | 7508 | -0.192 | -0.4669 | No |
| 217 | ACTN1 |  |  | 7520 | -0.193 | -0.4665 | No |
| 218 | CENPN |  |  | 7528 | -0.194 | -0.4657 | No |
| 219 | CMC2 |  |  | 7539 | -0.196 | -0.4652 | No |
| 220 | HPCAL1 |  |  | 7544 | -0.196 | -0.4640 | No |
| 221 | TPX2 |  |  | 7549 | -0.197 | -0.4627 | No |
| 222 | UBE2T |  |  | 7561 | -0.199 | -0.4623 | No |
| 223 | CDC42EP3 |  |  | 7562 | -0.199 | -0.4606 | No |
| 224 | BEX4 |  |  | 7566 | -0.199 | -0.4593 | No |
| 225 | BIRC5 |  |  | 7596 | -0.204 | -0.4609 | No |
| 226 | ACOT7 |  |  | 7623 | -0.207 | -0.4621 | No |
| 227 | CARHSP1 |  |  | 7667 | -0.213 | -0.4653 | No |
| 228 | DCXR |  |  | 7713 | -0.220 | -0.4687 | No |
| 229 | RAB9A |  |  | 7740 | -0.224 | -0.4698 | No |
| 230 | RND2 |  |  | 7823 | -0.239 | -0.4773 | Yes |
| 231 | MZT1 |  |  | 7838 | -0.242 | -0.4768 | Yes |
| 232 | FDFT1 |  |  | 7852 | -0.244 | -0.4762 | Yes |
| 233 | PNRC1 |  |  | 7867 | -0.247 | -0.4758 | Yes |
| 234 | CENPU |  |  | 7871 | -0.248 | -0.4740 | Yes |
| 235 | RBP1 |  |  | 7877 | -0.250 | -0.4724 | Yes |
| 236 | DHFR |  |  | 7878 | -0.250 | -0.4703 | Yes |
| 237 | PSME2 |  |  | 7885 | -0.252 | -0.4689 | Yes |
| 238 | ZFAND6 |  |  | 7909 | -0.257 | -0.4693 | Yes |
| 239 | EIF1B |  |  | 7931 | -0.262 | -0.4695 | Yes |
| 240 | SOX4 |  |  | 7958 | -0.268 | -0.4703 | Yes |
| 241 | H2AFX |  |  | 7965 | -0.269 | -0.4687 | Yes |
| 242 | CKS2 |  |  | 7972 | -0.270 | -0.4671 | Yes |
| 243 | HMGB2 |  |  | 7973 | -0.271 | -0.4647 | Yes |
| 244 | HPRT1 |  |  | 7974 | -0.271 | -0.4624 | Yes |
| 245 | CENPV |  |  | 8000 | -0.276 | -0.4630 | Yes |
| 246 | TPM1 |  |  | 8012 | -0.278 | -0.4619 | Yes |
| 247 | CITED1 |  |  | 8014 | -0.278 | -0.4596 | Yes |
| 248 | EZR |  |  | 8030 | -0.281 | -0.4590 | Yes |
| 249 | DECR1 |  |  | 8034 | -0.282 | -0.4569 | Yes |
| 250 | TAGLN3 |  |  | 8037 | -0.283 | -0.4547 | Yes |
| 251 | DTYMK |  |  | 8075 | -0.292 | -0.4565 | Yes |
| 252 | CTNNAL1 |  |  | 8076 | -0.292 | -0.4540 | Yes |
| 253 | CDKN3 |  |  | 8079 | -0.293 | -0.4518 | Yes |
| 254 | PCNA |  |  | 8082 | -0.293 | -0.4495 | Yes |
| 255 | NLRP1 |  |  | 8106 | -0.298 | -0.4496 | Yes |
| 256 | DUT |  |  | 8118 | -0.300 | -0.4483 | Yes |
| 257 | CCT5 |  |  | 8125 | -0.302 | -0.4465 | Yes |
| 258 | RPS27L |  |  | 8126 | -0.302 | -0.4439 | Yes |
| 259 | SAT1 |  |  | 8162 | -0.314 | -0.4453 | Yes |
| 260 | MRPL15 |  |  | 8184 | -0.321 | -0.4450 | Yes |
| 261 | SRSF3 |  |  | 8191 | -0.323 | -0.4429 | Yes |
| 262 | TUBB2B |  |  | 8224 | -0.332 | -0.4438 | Yes |
| 263 | CKS1B |  |  | 8241 | -0.336 | -0.4428 | Yes |
| 264 | HOPX |  |  | 8257 | -0.341 | -0.4416 | Yes |
| 265 | LDHA |  |  | 8263 | -0.343 | -0.4393 | Yes |
| 266 | PPA1 |  |  | 8265 | -0.344 | -0.4364 | Yes |
| 267 | UBE2C |  |  | 8278 | -0.348 | -0.4349 | Yes |
| 268 | CNP |  |  | 8294 | -0.352 | -0.4336 | Yes |
| 269 | DCTPP1 |  |  | 8303 | -0.356 | -0.4315 | Yes |
| 270 | EIF4EBP1 |  |  | 8306 | -0.356 | -0.4287 | Yes |
| 271 | MRPL52 |  |  | 8318 | -0.359 | -0.4269 | Yes |
| 272 | PA2G4 |  |  | 8320 | -0.360 | -0.4239 | Yes |
| 273 | SOX9 |  |  | 8327 | -0.361 | -0.4216 | Yes |
| 274 | EIF1AX |  |  | 8331 | -0.363 | -0.4188 | Yes |
| 275 | TSC22D1 |  |  | 8334 | -0.363 | -0.4159 | Yes |
| 276 | CAP1 |  |  | 8335 | -0.363 | -0.4129 | Yes |
| 277 | CCND1 |  |  | 8346 | -0.368 | -0.4109 | Yes |
| 278 | GCHFR |  |  | 8348 | -0.369 | -0.4078 | Yes |
| 279 | TUBB4B |  |  | 8356 | -0.371 | -0.4055 | Yes |
| 280 | NUDT1 |  |  | 8359 | -0.373 | -0.4025 | Yes |
| 281 | CLIC1 |  |  | 8368 | -0.378 | -0.4002 | Yes |
| 282 | GTF3A |  |  | 8399 | -0.388 | -0.4004 | Yes |
| 283 | SH3BGRL |  |  | 8409 | -0.390 | -0.3981 | Yes |
| 284 | NDUFA12 |  |  | 8414 | -0.392 | -0.3952 | Yes |
| 285 | HSPD1 |  |  | 8418 | -0.393 | -0.3922 | Yes |
| 286 | HMGB3 |  |  | 8427 | -0.399 | -0.3897 | Yes |
| 287 | ACAT2 |  |  | 8440 | -0.404 | -0.3877 | Yes |
| 288 | PTTG1 |  |  | 8446 | -0.406 | -0.3848 | Yes |
| 289 | FEZ1 |  |  | 8457 | -0.412 | -0.3825 | Yes |
| 290 | ITGB1BP1 |  |  | 8459 | -0.413 | -0.3790 | Yes |
| 291 | PFDN2 |  |  | 8465 | -0.417 | -0.3761 | Yes |
| 292 | CCT6A |  |  | 8469 | -0.418 | -0.3728 | Yes |
| 293 | LYPD1 |  |  | 8476 | -0.422 | -0.3699 | Yes |
| 294 | LMNA |  |  | 8478 | -0.423 | -0.3664 | Yes |
| 295 | CACYBP |  |  | 8486 | -0.428 | -0.3636 | Yes |
| 296 | TPM3 |  |  | 8487 | -0.428 | -0.3600 | Yes |
| 297 | ZCCHC17 |  |  | 8492 | -0.430 | -0.3567 | Yes |
| 298 | SNRNP25 |  |  | 8494 | -0.432 | -0.3532 | Yes |
| 299 | TMEM158 |  |  | 8499 | -0.434 | -0.3499 | Yes |
| 300 | POLD2 |  |  | 8508 | -0.439 | -0.3471 | Yes |
| 301 | ST13 |  |  | 8512 | -0.442 | -0.3437 | Yes |
| 302 | SNRPD1 |  |  | 8537 | -0.451 | -0.3426 | Yes |
| 303 | PFN2 |  |  | 8545 | -0.455 | -0.3396 | Yes |
| 304 | C12orf75 |  |  | 8546 | -0.456 | -0.3357 | Yes |
| 305 | LGALS3 |  |  | 8550 | -0.460 | -0.3321 | Yes |
| 306 | TAGLN2 |  |  | 8553 | -0.461 | -0.3284 | Yes |
| 307 | WDR1 |  |  | 8554 | -0.463 | -0.3244 | Yes |
| 308 | RAN |  |  | 8555 | -0.463 | -0.3205 | Yes |
| 309 | RPA3 |  |  | 8572 | -0.471 | -0.3183 | Yes |
| 310 | NME1 |  |  | 8573 | -0.471 | -0.3143 | Yes |
| 311 | PRDX1 |  |  | 8584 | -0.476 | -0.3114 | Yes |
| 312 | RAB13 |  |  | 8590 | -0.481 | -0.3079 | Yes |
| 313 | TYMS |  |  | 8599 | -0.488 | -0.3046 | Yes |
| 314 | ZNF428 |  |  | 8602 | -0.493 | -0.3007 | Yes |
| 315 | HMGN2 |  |  | 8608 | -0.496 | -0.2970 | Yes |
| 316 | COTL1 |  |  | 8612 | -0.501 | -0.2931 | Yes |
| 317 | METRN |  |  | 8638 | -0.518 | -0.2916 | Yes |
| 318 | CYCS |  |  | 8640 | -0.519 | -0.2873 | Yes |
| 319 | HMGB1 |  |  | 8653 | -0.527 | -0.2841 | Yes |
| 320 | SIVA1 |  |  | 8654 | -0.529 | -0.2796 | Yes |
| 321 | MARCKS |  |  | 8667 | -0.537 | -0.2764 | Yes |
| 322 | ARPC5 |  |  | 8669 | -0.540 | -0.2719 | Yes |
| 323 | PRDX6 |  |  | 8673 | -0.540 | -0.2677 | Yes |
| 324 | HMGA1 |  |  | 8675 | -0.543 | -0.2632 | Yes |
| 325 | ID3 |  |  | 8676 | -0.544 | -0.2585 | Yes |
| 326 | SLC25A5 |  |  | 8681 | -0.546 | -0.2543 | Yes |
| 327 | SNRPB |  |  | 8683 | -0.547 | -0.2498 | Yes |
| 328 | DRAP1 |  |  | 8691 | -0.552 | -0.2459 | Yes |
| 329 | MLLT11 |  |  | 8695 | -0.555 | -0.2415 | Yes |
| 330 | C4orf3 |  |  | 8705 | -0.565 | -0.2377 | Yes |
| 331 | ARPC2 |  |  | 8725 | -0.586 | -0.2349 | Yes |
| 332 | YWHAH |  |  | 8726 | -0.587 | -0.2299 | Yes |
| 333 | PDCD5 |  |  | 8729 | -0.592 | -0.2251 | Yes |
| 334 | ENO1 |  |  | 8734 | -0.593 | -0.2205 | Yes |
| 335 | S100A16 |  |  | 8739 | -0.596 | -0.2159 | Yes |
| 336 | CALM2 |  |  | 8744 | -0.600 | -0.2112 | Yes |
| 337 | TUBB |  |  | 8763 | -0.621 | -0.2080 | Yes |
| 338 | ATP6V1G1 |  |  | 8766 | -0.625 | -0.2029 | Yes |
| 339 | DEK |  |  | 8770 | -0.627 | -0.1979 | Yes |
| 340 | H2AFZ |  |  | 8774 | -0.630 | -0.1929 | Yes |
| 341 | STMN1 |  |  | 8783 | -0.638 | -0.1883 | Yes |
| 342 | FABP7 |  |  | 8811 | -0.661 | -0.1858 | Yes |
| 343 | MZT2B |  |  | 8816 | -0.665 | -0.1806 | Yes |
| 344 | RANBP1 |  |  | 8819 | -0.669 | -0.1751 | Yes |
| 345 | BEX1 |  |  | 8833 | -0.689 | -0.1708 | Yes |
| 346 | PRELID1 |  |  | 8847 | -0.705 | -0.1663 | Yes |
| 347 | HIST1H4C |  |  | 8850 | -0.710 | -0.1604 | Yes |
| 348 | LSM4 |  |  | 8853 | -0.712 | -0.1546 | Yes |
| 349 | MARCKSL1 |  |  | 8857 | -0.716 | -0.1488 | Yes |
| 350 | ACTG1 |  |  | 8859 | -0.718 | -0.1428 | Yes |
| 351 | H3F3A |  |  | 8867 | -0.728 | -0.1374 | Yes |
| 352 | TUBA1A |  |  | 8883 | -0.751 | -0.1327 | Yes |
| 353 | PTMA |  |  | 8894 | -0.764 | -0.1274 | Yes |
| 354 | CHCHD2 |  |  | 8898 | -0.770 | -0.1212 | Yes |
| 355 | PPP1R14B |  |  | 8900 | -0.771 | -0.1147 | Yes |
| 356 | UBE2S |  |  | 8901 | -0.772 | -0.1081 | Yes |
| 357 | TPM4 |  |  | 8905 | -0.778 | -0.1018 | Yes |
| 358 | S100B |  |  | 8908 | -0.784 | -0.0954 | Yes |
| 359 | ACTB |  |  | 8912 | -0.793 | -0.0889 | Yes |
| 360 | TXN |  |  | 8952 | -0.885 | -0.0859 | Yes |
| 361 | CSRP2 |  |  | 8954 | -0.891 | -0.0784 | Yes |
| 362 | EEF1A1 |  |  | 8958 | -0.915 | -0.0710 | Yes |
| 363 | PKM |  |  | 8959 | -0.915 | -0.0631 | Yes |
| 364 | S100A10 |  |  | 8965 | -0.932 | -0.0558 | Yes |
| 365 | RPSA |  |  | 8973 | -1.005 | -0.0480 | Yes |
| 366 | PFN1 |  |  | 8977 | -1.045 | -0.0394 | Yes |
| 367 | TUBA1B |  |  | 8980 | -1.103 | -0.0303 | Yes |
| 368 | FTH1 |  |  | 8981 | -1.131 | -0.0206 | Yes |
| 369 | TMSB4X |  |  | 8983 | -1.142 | -0.0110 | Yes |
| 370 | FTL |  |  | 8984 | -1.286 | -0.0000 | Yes |
Table: GSEA details [plain text format]

  

Fig 2: FELDMAN\_2020\_NEURAL G0: Random ES distribution      
 Gene set null distribution of ES for **FELDMAN\_2020\_NEURAL G0**
